# Evolutionarily conserved enhancer-associated features within the *MYEOV* locus suggest a regulatory role for this non-coding DNA region in cancer

**DOI:** 10.3389/fcell.2024.1294510

**Published:** 2024-07-30

**Authors:** Brigid S. A. Davidson, Juliana Estefania Arcila-Galvis, Marco Trevisan-Herraz, Aneta Mikulasova, Chris A. Brackley, Lisa J. Russell, Daniel Rico

**Affiliations:** ^1^ Biosciences Institute, Newcastle University, Newcastle upon Tyne, United Kingdom; ^2^ SUPA, School of Physics and Astronomy, University of Edinburgh, Edinburgh, United Kingdom; ^3^ CABIMER, CSIC-Universidad de Sevilla-Universidad Pablo de Olavide-Junta de Andalucía, Seville, Spain

**Keywords:** enhancer, evolution, oncogene, *MYEOV*, *CCND1* (Cyclin D1)

## Abstract

The *myeloma overexpressed* gene (*MYEOV*) has been proposed to be a proto-oncogene due to high RNA transcript levels found in multiple cancers, including myeloma, breast, lung, pancreas and esophageal cancer. The presence of an open reading frame (ORF) in humans and other primates suggests protein-coding potential. Yet, we still lack evidence of a functional MYEOV protein. It remains undetermined how *MYEOV* overexpression affects cancerous tissues. In this work, we show that *MYEOV* has likely originated and may still function as an enhancer, regulating *CCND1* and *LTO1*. Firstly, *MYEOV* 3′ enhancer activity was confirmed in humans using publicly available ATAC-STARR-seq data, performed on B-cell-derived GM12878 cells. We detected enhancer histone marks H3K4me1 and H3K27ac overlapping *MYEOV* in multiple healthy human tissues, which include B cells, liver and lung tissue. The analysis of 3D genome datasets revealed chromatin interactions between a *MYEOV-3*′*-putative enhancer* and the proto-oncogene *CCND1*. BLAST searches and multi-sequence alignment results showed that DNA sequence from this human enhancer element is conserved from the amphibians/amniotes divergence, with a 273 bp conserved region also found in all mammals, and even in chickens, where it is consistently located near the corresponding *CCND1* orthologues. Furthermore, we observed conservation of an active enhancer state in the *MYEOV* orthologues of four non-human primates, dogs, rats, and mice. When studying this homologous region in mice, where the ORF of *MYEOV* is absent, we not only observed an enhancer chromatin state but also found interactions between the mouse enhancer homolog and *Ccnd1* using 3D-genome interaction data. This is similar to the interaction observed in humans and, interestingly, coincides with CTCF binding sites in both species. Taken together, this suggests that *MYEOV* is a primate-specific gene with a *de novo* ORF that originated at an evolutionarily older enhancer region. This deeply conserved putative enhancer element could regulate *CCND1* in both humans and mice, opening the possibility of studying *MYEOV* regulatory functions in cancer using non-primate animal models.

## 1 Introduction

*MYEOV* (*myeloma overexpressed*) is a gene believed to be present only in primates (Papamichos et al., 2015). It is proposed to be a proto-oncogene, as its RNA expression has been linked to poorer prognosis in multiple cancers. Cancers showing overexpression of *MYEOV* transcripts include multiple myeloma (Moreaux et al., 2010), breast cancer (Janssen et al., 2002a), esophageal squamous cell carcinomas (Janssen et al., 2002b), gastric cancer (Leyden et al., 2006), colon cancer (Moss et al., 2006), neuroblastoma (Takita et al., 2011) and, more recently, pancreatic ductal adenocarcinoma (PDAC) (Fang et al., 2019; Liang et al., 2020) and non-small cell lung cancer (NSCLC) (Fang et al., 2019). *MYEOV* has also been proposed as a biomarker for prognosis in hepatocellular carcinoma (Deng et al., 2019). However, the exact role that *MYEOV* has in both healthy and cancerous cells remains elusive.

The function of *MYEOV* in healthy cells is currently understudied with competing evidence as to whether it is expressed at very low levels in certain healthy tissues (Liang et al., 2020), with no detectable protein expression in others (Fang et al., 2019). *MYEOV* is believed to have originated with a *de novo* open reading frame (ORF) acquisition during the Catarrhini/Platyrrhini divergence *via* a mutation leading to the *MYEOV*-313 start codon (Papamichos et al., 2015). Interestingly, *MYEOV*’s protein coding potential is thought to be found only in humans and not in other primates, probably due to a human-specific mutation leading to acquisition of a start codon upstream of the *MYEOV*-255 start codon, extending *MYEOV*’s ORF (Papamichos et al., 2015).

In cancer, it has been shown that the increased RNA expression of *MYEOV* can be caused by genomic rearrangements or duplications of 11q13 (Janssen et al., 2002b; Liang et al., 2020) or by hypomethylation of *MYEOV*’s promoter (Fang et al., 2019; Liang et al., 2020). In PDAC, it was reported that MYEOV protein interacts with the oncogenic transcription factor SOX9 (Liang et al., 2020). MYEOV has been associated with the regulation of microRNAs miR-17-5p and miR-93-5p, possibly by interacting with MYC (Shen et al., 2021). Knockdown of *MYEOV* in pancreatic cell lines suppresses expression of folate metabolic enzymes such as MTHFD2 and restores expression of MYC and mTORC1 repressors (Tange et al., 2023). In NSCLC, it has been proposed to act as a competing endogenous RNA (ceRNA) where it would inhibit the activity of microRNA miR-30c-2-3p (Fang et al., 2019). In fact, a recent paper has also indicated that *MYEOV* might have a role as a ceRNA in PDAC mirroring results seen in NSCLC (Chen et al., 2022).

There is also debate over MYEOV protein function in cancer due to the presence of four upstream translation start sites, believed to render it impossible for *MYEOV* to be translated in human cells (de Almeida et al., 2006). These four upstream AUG sequences located in *MYEOV*’s 5′ untranslated region (UTR) would prevent ribosomal binding *via* regulation of the ribosomal entry site (Barbosa et al., 2013), abrogating the translation of the *MYEOV* transcript (de Almeida et al., 2006). More recently a paper reported MYEOV protein expression in PDAC, where they have shown both *via* immunohistochemistry and western blots that tumour cells exhibit MYEOV protein expression (Liang et al., 2020). This result has not been replicated in NSCLC cell lines where only *MYEOV* RNA expression was observed (Fang et al., 2019). It is possible that *MYEOV* translation might be dependent on cellular states as seen for proinsulin where translational control *via* upstream AUGs is affected by developmental stages (Hernández-Sánchez et al., 2005).

In our recent study, we explored the chromatin landscape of *MYEOV* in healthy B cells and discovered enhancer-like features within and 3′ end of the gene (Mikulasova et al., 2022). Our previous analyses suggest potential regulatory connections between *MYEOV* and the proto-oncogene *CCND1*. We observed that in the multiple myeloma (MM) cell line U266, the insertion of the *immunoglobulin heavy chain* (*IGH*) Eα1 super-enhancer upstream of *CCND1* not only changes the chromatin state surrounding *CCND1* but also alters the chromatin configuration of the *MYEOV* gene. We found increased chromosomal accessibility over the *MYEOV* gene body and a H3K4me3 broad domain covering most of the gene (Mikulasova et al., 2022). This is not the first time that *MYEOV* and *CCND1* have been associated together; in fact co-amplification of these two genes are seen in multiple cancers, in particular MM where a 11q13 duplication has occurred (Janssen et al., 2000; Janssen et al., 2002b). They have also been linked together in esophageal squamous cell carcinomas where co-amplification of both genes leads to epigenetic silencing of *MYEOV* (Janssen et al., 2000; Janssen et al., 2002b) and in primary plasma cell leukaemia where the t(11;14) chromosomal rearrangement leads to *IGH* super-enhancers being juxtaposed next to *MYEOV* and *CCND1* leading to overexpression of both genes (Coccaro et al., 2016). However, the possible role of *MYEOV* locus as an enhancer and the potential regulatory connections with *CCND1* in healthy cells have not been investigated.

In order to further elucidate the possible function of the *MYEOV* locus in healthy and cancer cells, we have integrated publicly available epigenomics, 3D genome and comparative genomics data to characterise the *MYEOV-3*′*-putative enhancer* region and elucidate its evolutionary origins. Our data strongly suggests that this genomic region is a regulatory element that is older than the ORF. The core putative enhancer DNA sequence is conserved across mammals (even if they lack the ORF), the *CCND1* homologue is frequently in synteny, showing 3D interactions with *MYEOV-3*′*-putative enhancer* region in human and mouse cells.

## 2 Material and methods

### 2.1 Quantification of enhancer activity *via* ATAC-STARR-seq

ATAC-STARR-seq data obtained from GM12878 (Wang X. et al., 2018) was used to confirm the enhancer activity of the *MYEOV-3*′*-putative enhancer*, measured by the ability of open chromatin regions within the *MYEOV* locus to self-transcribe. This was done by isolating open chromatin fragments using ATACseq, cloning these regions into the 3′UTR region of GFP protein plasmids and transfection of these fragments into target cells (in this case GM12878 cells). Regions with regulatory activity will then self-activate and be transcribed. Active regions were then defined by comparing RNA signal to DNA-library from non-transfected cells and were made available as BED files (the accession number of this dataset, together with the ones corresponding to the other datasets listed below, are listed in Supplementary Table S1).

### 2.2 Epigenomic datasets

#### 2.2.1 Epigenomic data from human cell types and tissues

ChIP-seq datasets for H3K4me1, H3K4me3, and H3K27ac histone modifications were retrieved as processed BigWig files from the NIH Epigenomic Roadmap (Bernstein et al., 2010) to determine the chromatin state surrounding *MYEOV* in human B cells, lung, liver and pancreas (Supplementary Table S1).

The entire catalogue (as of December 2021) of human derived H3K27ac ChIP-seq data on the ENCODE database (ENCODE Project Consortium, 2012) was used to determine in which tissues *MYEOV-3*′*-putative enhancer* region was active (Supplementary Table S2).

#### 2.2.2 Epigenomic data from other species

To determine chromatin state and sequence conservation of the *MYEOV*-*3*′-putative enhancer, we obtained ChIP-seq data for the five major regulatory histone marks including H3K4me1, H3K4me3, H3K27ac, H3K27me3, and H3K36me3, combined with ATAC-seq in lymphoblastoid cell lines of five primate species (human, chimpanzee, gorilla, orangutan and macaque) (García-Pérez et al., 2021); see details in Supplementary Table S1.

ChIP-seq for H3K4me1, H3K4me3, and H3K27ac histone modifications in CH12 mouse B cell lymphoma cell line, adult liver cells, lung cells, and bone marrow, alongside CTCF from B cells, were retrieved from ENCODE (ENCODE Project Consortium et al., 2020), see Supplementary Table S1.

We gathered available ChIP-seq data (H3K4me1, H3K4me3, and H3K27ac) derived from liver cells of five mammalian species (macaques, dogs, cats, mice, rats) from Roller *et al.* (Roller et al., 2021) (Supplementary Table S1) with H3K4me3 and H3K27ac ChIP-seq data from the liver of six mammalian species (human, mice, rats, dogs, bovine, and pigs) from Villar *et al.*, (Villar et al., 2015), and H3K4me3 and H3K27ac ChIP-seq data from the liver of mice (Shen et al., 2012) (Supplementary Table S1).

ChIP-seq datasets for histone marks H3K4me1, H3K4me3, and H3K27ac from chicken liver tissue (Kern et al., 2021) were used to determine chromatin state and sequence conservation of the *MYEOV-3*′*-putative enhancer*. See Supplementary Table S1.

#### 2.2.3 Defining *MYEOV*-3′-putative enhancer location

The human *MYEOV-3*′*-putative enhancer* was defined by active regions determined by ATAC-STAR-seq in GM12878, together with the presence of histone marks H3K27ac, H3K4me1 (with or without the presence of H3K4me3) and regions of DNase I hypersensitivity defined by DNase-seq. The *MYEOV-3*′*-putative enhancer* maps to chr11:69,297,041-69,298,856 (GRCh38/hg38), unless another region is specifically referred to.

#### 2.2.4 *MYEOV* gene expression data

RNA-seq datasets retrieved as processed BigWig files from ENCODE (ENCODE Project Consortium et al., 2020) to determine *MYEOV* expression in human B cells, lung, liver and pancreas (Supplementary Table S1).

### 2.3 Chromatin conformation data

#### 2.3.1 Chromatin interactions in human cells

Chromatin interaction data were used to determine the possible targets of the *MYEOV-3*′*-putative enhancer* element, using the data available at the 3D Genome Browser (Wang Y. et al., 2018). Identification of gene targets of the *MYEOV-3*′*-putative enhancer* was obtained using promoter capture Hi-C (PCHi-C) data, for a list of tissues used in this analysis see Supplementary Table S3. These cell types were chosen due to *MYEOV* expression being proposed as a prognostic factor for these tissue associated cancers (Moreaux et al., 2010; Fang et al., 2019; Shen et al., 2021). Chromatin Interaction Analysis with Paired-End-Tag sequencing (ChIA-PET) datasets were also obtained, see Supplementary Table S3. This was taken alongside CTCF data, Supplementary Table S3.

PCHi-C from pre-B cells (Koohy et al., 2018), chromatin capture Hi-C data from mouse embryonic cells (mESC) (Joshi et al., 2015), and PCHi-C data from mESC (Schoenfelder et al., 2015) were used to detect mouse *Ccnd1* promoter interactions with possible target enhancers, see Supplementary Table S3. In the pre-B cells only, interactions between 19 and 22 months mice (“old mice”) were included (Koohy et al., 2018).

#### 2.3.2 Hi-C and HiP-HoP simulations in GM12878 cells

We used the high resolution Hi-C data from Rao *et al.* (Rao et al., 2014) and predicted interactions derived using the HiP-HoP model (Buckle et al., 2018). This modelling approach uses histone modification and DNase I hypersensitivity data to generate a population of 3D chromatin configurations from which simulated Hi-C data can be obtained. The input data and model parameters have been described in detail in our previous study (Rico et al., 2022).

### 2.4 Visualisation of epigenomic and chromatin conformation data

The WashU Epigenome Browser (Li et al., 2022) (http://epigenomegateway.wustl.edu/browser), the 3D Genome Browser (http://3dgenome.fsm.northwestern.edu) (Wang Y. et al., 2018), Ensembl (v104) website (Martin et al., 2023) and the Integrative Genomics Viewer, IGV (Robinson et al., 2011) were used for data visualisation.

### 2.5 Evolutionary analysis of the DNA sequence of *MYEOV-3*′*-putative enhancer*

To determine each individual species’ possible enhancer DNA sequence, BLASTN searches were performed using the Ensembl (v104) website (Martin et al., 2023). Briefly, the DNA sequence of the *MYEOV* human enhancer element, defined by (García-Pérez et al., 2021). From lymphoblastoid cell lines using ChromHMM, GRCh38/hg38 chr11:69,293,400-69,300,600 (García-Pérez et al., 2021), was taken from Ensembl and blasted against 17 species (see full list in Supplementary Table S4). This DNA region was selected as it encompassed both *MYEOV*’s 3′UTR region and gene body in humans and all non-human primates. Search sensitivity for BLAST was set to distant homologies and Maximum E-value in which a hit would be reported was set to 1, the rest of the parameters were left as by default. Multiple sequence alignment was completed using Mauve with default parameters (Darling et al., 2004). From this alignment, we found the exact region conserved across the different species (Supplementary Table S4). Sequences in this region were realigned using the Clustal Omega alignment tool with default parameters (Sievers and Higgins, 2021). This alignment was manually curated using MEGA (Stecher et al., 2020).

## 3 Results

### 3.1 *MYEOV*’s 3′ region has transcriptional enhancer activity in human cells

Chromatin state data from the BLUEPRINT Consortium (Carrillo-de-Santa-Pau et al., 2017) showed that the entirety of *MYEOV*’*s* gene body is encompassed by enhancer chromatin states in healthy B cells, extending to the 3′ region of *MYEOV* (Mikulasova et al., 2022). This enhancer region was defined by the presence of histone marks H3K4me1 and H3K27ac, with or without the presence of H3K4me3.

**FIGURE 1:**
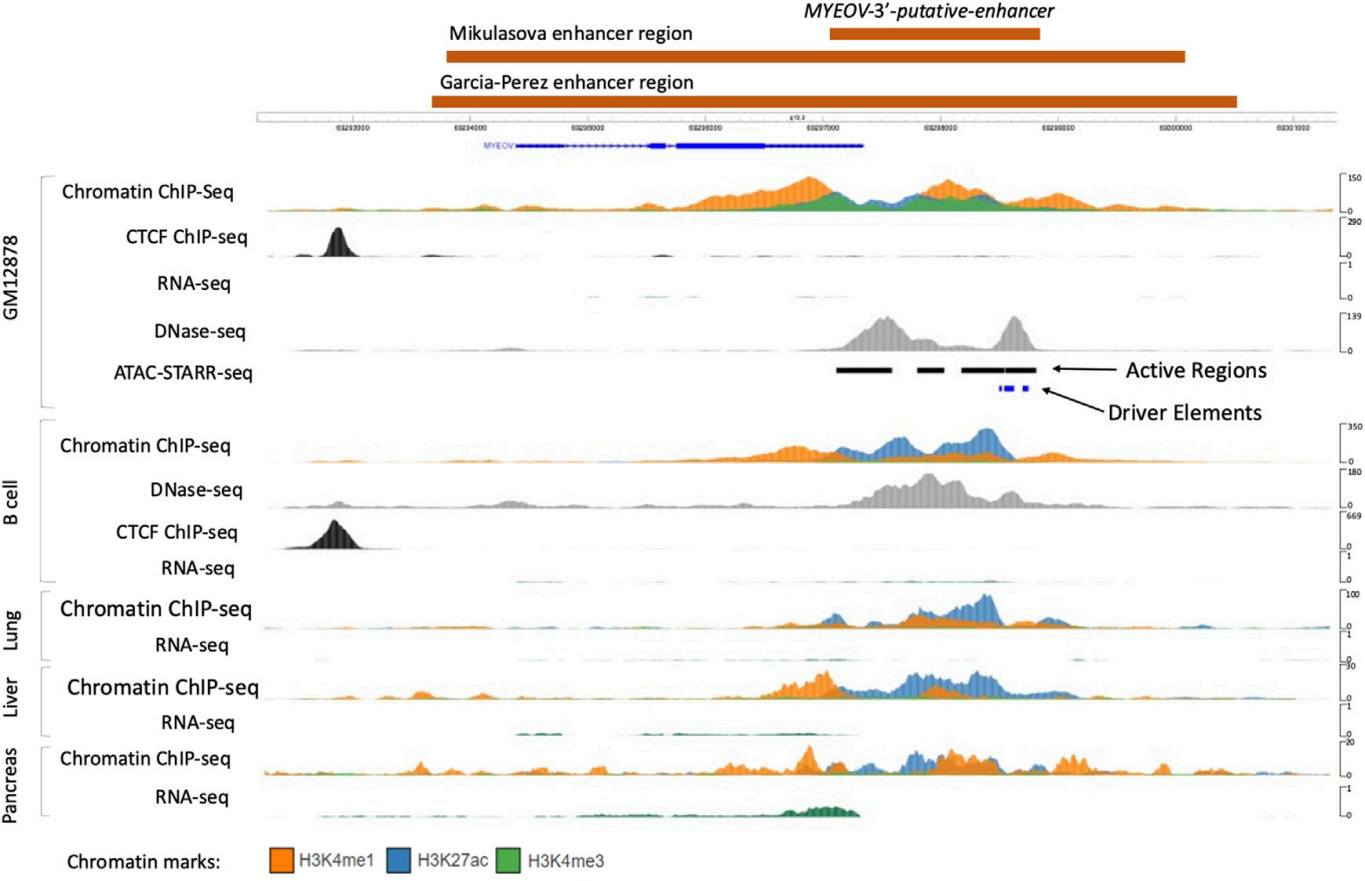
Location of the *MYEOV-3*′*-putative enhancer*. ChIP-seq, RNAseq and DNase-Seq data from lymphoblastoid cell line GM12878 were accessed from the ENCODE dataset alongside active regions and driver elements identified using the SHARPR-RE method (Wang X. et al., 2018), which predicts sub-regions with high regulatory activity at nucleotide resolution. *MYEOV*-3′-*putative enhancer*, Mikulasova (Mikulasova et al., 2022) and Garcia-Perez (García-Pérez et al., 2021) regulatory elements are all shown in orange blocks. ChIP-seq and RNAseq data from B cell, lung, liver and pancreatic tissue together with DNase-seq data in B cells, taken from the ENCODE dataset and visualised using the WashU Epigenome Browser.

To investigate *MYEOV*’s possible activity as a transcriptional enhancer, we used ATAC-STARR-seq data generated from the GM12878 lymphoblastoid B cell line (Wang X. et al., 2018). ATAC-STARR-seq is based on ATAC-seq isolation of open chromatin regions which are then inserted into a reporter gene within a plasmid, before being transfected into GM12878 cells. If tested open chromatin regions have a regulatory function, they will self-activate, leading to transcription of the reporter gene, measured using RNAseq (Arnold et al., 2013). This experimental approach allows for *in vitro* identification of open chromatin regions which have regulatory functions. Using this method, over 65,000 regulatory regions (active regions) were reported in GM12878 cells, four of which were located in the 3′ UTR region of *MYEOV* (Figure 1). We observed that these regions align with H3K27ac and H3K4me1 peaks and with DNase I hypersensitivity sites in GM12878 cells (Figure 1). Together these observations suggest that *MYEOV*’*s* 3′ UTR and the 3′ flanking region could have an enhancer function and from now on, we will refer to this region as the human *MYEOV-3*′*-putative enhancer* (see Materials and Methods and Figure 1). This region is smaller than the regulatory element defined, using histone marks, both by us in a previous work (Mikulasova et al., 2022) or by other researchers (García-Pérez et al., 2021). In contrast, here we used the presence of histone marks and ATAC-STARR-seq active regions to more precisely map the core region of this putative enhancer. Interestingly, the histone mark profiles in the region do not indicate that *MYEOV* has an active promoter (Figure 1) with the absence of a sharp H3K4me3 peak at the transcriptional start site (TSS). From this, we next wanted to determine if there are any other cell types in which an equivalent putative enhancer is found overlapping *MYEOV*’s 3′ UTR region.

### 3.2 *MYEOV-3*′*-putative enhancer* region is active in multiple human cell types and tissues

First, we confirmed that the active enhancer chromatin state that was observed in B cells (Mikulasova et al., 2022) using BLUEPRINT data (Carrillo-de-Santa-Pau et al., 2017) could be validated with other datasets. To do this, we used ChIP-seq data for three histone marks (H3K4me1, H3K4me3, and H3K27ac) from the NIH Roadmap (Roadmap Epigenomics Consortium et al., 2015) and ENCODE (ENCODE Project Consortium, 2012) consortia. In the ENCODE datasets we found that the *MYEOV* locus indeed exhibited an enhancer chromatin state in B cells, denoted by the presence of H3K4me1 and H3K27ac (Figure 1). This result is mirrored in data from the NIH Roadmap Consortium, where H3K4me1 and H3K27ac peaks were observed in B cells at the *MYEOV* locus (Supplementary Figure S1). This enhancer chromatin state was primarily located in the *MYEOV*’s 3′ UTR region, the same location as observed in GM12878 cells (Figures 1A,B; Supplementary Figure S1). Again confirming this as the location of this putative enhancer.

While the enhancer element is primarily located in *MYEOV*’s 3′ UTR, H3K4me1 is observed across the gene body of *MYEOV* in B cells (Figure 1; Supplementary Figure S1). No active promoter state can be seen over the TSS of *MYEOV* as evidenced by the absence of H3K4me3 and H3K27ac sharp peaks in this region, suggesting that *MYEOV* enhancer activity might be independent of *MYEOV* gene transcription in healthy B cells (Figure 1; Supplementary Figure S1). This is consistent with the complete lack of RNA-seq signals in this region (Figure 1), suggesting that in B cells the primary function of this region could be to act as an enhancer element.

Next, we expanded our search to determine if the *MYEOV-3*′*-putative enhancer* was active in other tissues. We started with three tissues where *MYEOV* expression had previously been linked to cancer including lung (Fang et al., 2019), liver (Deng et al., 2019), and pancreatic tissue (Fang et al., 2019; Shen et al., 2021). In both, the ENCODE (Figure 1) and NIH Epigenomics Roadmap data (Supplementary Figure S1A), H3K4me1 and H3K27ac signals were observed in *MYEOV-3*′*-putative enhancer*, highlighting that the histone marks associated with active enhancer state are present in this area. Just as in B cells, these cell types showed no active promoter chromatin state at *MYEOV*’s TSS and again no RNA transcripts were observed in this region for lung and liver cells, with only low level transcription observed in pancreatic cells. Interestingly, this signal also tends to co-localise in the *MYEOV-3*′*-putative enhancer*. Therefore the lack of promoter chromatin signals suggests that *MYEOV*-3′-*putative enhancer* activity is independent of *MYEOV*’s gene promoter activity (Figure 1; Supplementary Figure S1).

Using the 182 samples from ENCODE, including primary cells, tissues and cell line samples, we discovered that 131 samples contained H3K27ac peaks within *MYEOV*’s 3′ UTR region (Supplementary Figure S1B; Supplementary Table S2), further supporting an enhancer role for this region in humans. Again, the presence of the active chromatin state surrounding *MYEOV* was associated with tissues in which increased expression is commonly associated with the onset of cancer such as liver, pancreas, colonic mucosa, lung, breast epithelium and stomach smooth muscle (Supplementary Table S2). No H3K27ac peaks were observed in 25 tissues, a group that includes brain tissues/primary cells, ovary and testis (Supplementary Table S2). To further elucidate this enhancer function and its potential role in cancer development, we aimed to identify possible target genes.

### 3.3 *MYEOV-3*′*-putative enhancer* interacts with *CCND1* in human

**FIGURE 2:**
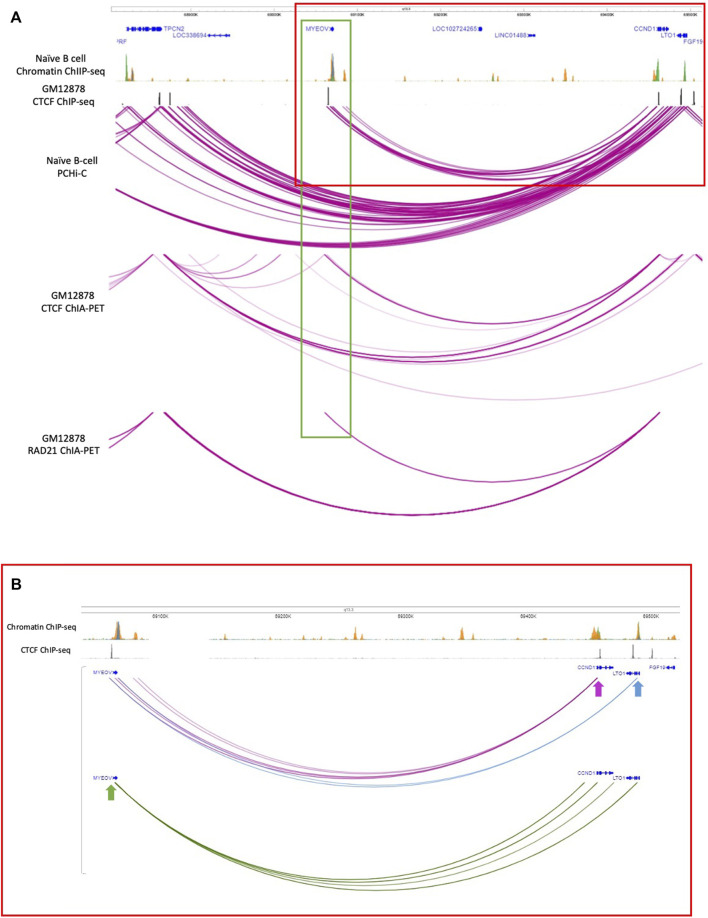
Interacting partners of the *MYEOV*-3′-putative *enhancer*. **(A)** Top panels: genome tracks showing ChIP-seq data for H3K4me1 (orange), H3K4me3 (green) and H3K27ac (blue) from primary B cells, and CTCF ChIP-seq data from GM12878 cells. Bottom panels: PCHi-C data from naive B cells, where the purple arcs denote interactions between fragments inside the TAD region containing *CCND1* and *MYEOV*. CTCF ChIA-PET data and RAD-21 ChIA-PET data in GM12878 cells with replicates combined. The purple arcs denote interactions between fragments. Data is visualised using the WashU Epigenome Browser. **(B)** Detail of interactions between *MYEOV*, *CCND1* and *LTO1* (for the region indicated with a red rectangle in panel A). Interactions where *CCND1* promoter was taken as the bait are highlighted in purple. Interactions where the LTO1 promoter was taken as bait are shown in blue. Interactions where *MYEOV* promoter was taken as bait are highlighted in green. Arrows indicate the baits. Data is visualised using the WashU Epigenome Browser.

To determine possible targets of the *MYEOV-3*′*-putative enhancer*, we analysed promoter-centred interaction data (PCHi-C) generated in both naive and total B cells (Javierre et al., 2016), where this putative enhancer element was originally discovered. Here, we observed that in naive B cells, *MYEOV*’s promoter region, when taken as the viewpoint (green box in Figure 2A), interacts with the promoters of *CCND1* and a gene located downstream, *LTO1* (previously known as *ORAVO1*). When the fragment baits containing the promoters of *CCND1* (pink arrow in Figure 2B) and *LTO1* (blue arrow in Figure 2B) were taken as the viewpoints, interactions with the *MYEOV*-3′-putative enhancer were observed. This suggests that in naive B cells, there are two possible targets of this putative enhancer element.

We then investigated whether these interactions were present in other primary cell types. We analysed PCHi-C data from primary tissues and cell lines in which we had not only shown an active enhancer chromatin state in *MYEOV*’s 3′ UTR and 3′ flanking region (Figure 1) but also with documented *MYEOV* overexpression association with cancer (Jung et al., 2019) (see Supplementary Figure S2). The resolution of PCHi-C data was not good enough to determine if the interactions were mediated *via MYEOV* promoter element or *MYEOV*-3′-putative enhancer, therefore we could only determine if the *MYEOV* region interacts with either gene. We observed interactions between the *MYEOV* region and the *CCND1* promoter-containing region in liver, lung, H1-derived mesenchymal stem cells (H1 cells) and the GM12878/GM19240 lymphoblastoid cell line (Supplementary Figure S2; Supplementary Table S5). This further supports our hypothesis that *MYEOV* interacts with and could regulate *CCND1*. Unexpectedly, no interactions between *MYEOV* and *CCND1* were seen in pancreatic tissues, despite the presence of histone marks denoting enhancer function surrounding *MYEOV* in this tissue type (Supplementary Figures S1–S3; Figure 1).

Given the observed interaction between *MYEOV* and *LTO1* in naive B cells, we used the *LTO1* promoter-containing region as another viewpoint (Supplementary Figure S4; Supplementary Table S5) and only found interactions with *MYEOV-3*′-putative enhancer in lymphoid cells (GM12878/GM1924), lung and H1 cells. When *MYEOV*’s promoter-containing region was used as the viewpoint, an interaction with *LTO1* was observed in lymphoid cells (GM12878/GM1924), H1 and liver cells (Supplementary Figures S3, S4; Supplementary Table S5). This suggests that *MYEOV-3*′*-putative enhancer* might have two possible targets, *CCND1* and *LTO1*, and that it targets them differently in different tissues.

To further strengthen this observation, we analysed ChIA-PET data to determine whether the interaction was mediated *via* CTCF cohesin loops as we had observed CTCF binding just upstream of *MYEOV* and downstream of *CCND1* in B cells. We used CTCF ChIA-PET data from the GM12878 cell line (ENCODE Project Consortium, 2012), where interactions between *MYEOV* and *CCND1* had already been detected (Supplementary Figures S2, S3). We found interactions between the CTCF site located upstream of *MYEOV* and *CCND1*, suggesting these two genes might interact using CTCF cohesin looping (Figures 2A,B). Interestingly, we did not see interactions between *CCND1* and *MYEOV*-3′*-putative enhancer* which is downstream of the CTCF site (Figure 2A). We did observe interactions between the CTCF site immediately upstream of *MYEOV* and another CTCF site located further upstream of *MYEOV*, close to *TPCN2*, suggesting CTCF-CTCF dimers and cohesin could be contributing to these interactions within this topologically associated domain (TAD) (Figure 2A). No interaction was seen between the CTCF site upstream of *MYEOV* and a CTCF site located close to *LTO1* (Figure 2A). To further validate that the interaction between *MYEOV* and *CCND1* is mediated by a CTCF/cohesin complex, we observed the same interaction between the CTCF site upstream of *MYEOV* and the *CCND1* CTCF site using ChIA-PET data derived from cohesin complex subunit, RAD21 (Figure 2A). Notably, we did not see any interaction between the CTCF site upstream of *MYEOV* and *TPCN2* CTCF sites (Figure 2A). However, we observed interactions between the CTCF sites of *CCND1* and TPCN2, a more distal gene that is located at the other boundary of the same TAD (Figure 2A). This suggests that the interaction between *MYEOV* and *CCND1* is mediated by a CTCF/cohesin complex not only in GM12878 cells but also in primary B cells (Figure 2A).

A more holistic view of chromatin interactions (i.e., not requiring enrichment through promoter capture or immunoprecipitation) can be obtained from Hi-C data, but these libraries often lack sufficient resolution needed to reveal enhancer-promoter interactions. An alternative approach is to use modelling schemes to predict interactions based on other types of epigenomic linear data (Buckle et al., 2018). We recently employed the highly-predictive heteromorphic polymer (HiP-HoP) simulation scheme to study the *CCND1* locus in B cell derived cell lines (Rico et al., 2022), and have previously shown that HiP-HoP provides accurate predictions when compared with high-resolution Capture-C data (Buckle et al., 2018; Rico et al., 2022).

**FIGURE 3:**
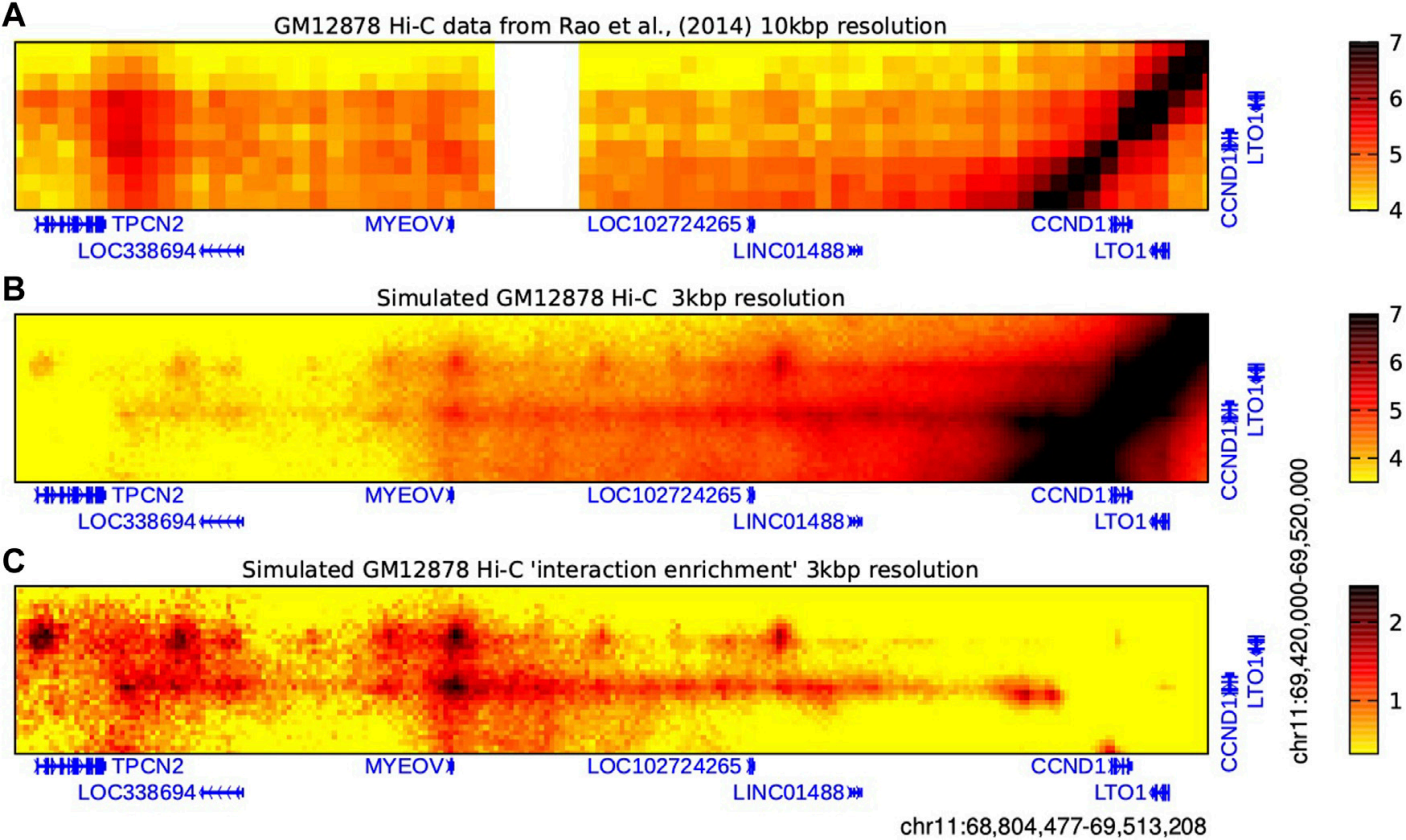
*MYEOV* 3D chromatin interactions evidenced by Hi-C data and HiP-HoP polymer simulations. Heatmaps showing real and simulated Hi-C data from GM12878 cells which reveal interactions between a broad region around *MYEOV* and a smaller region enclosing just *CCND1* and *LTO1*, as indicated. **(A)** Real Hi-C data are binned at 10 kbp resolution (Rao et al., 2014). Colour scale units are log-normalised interaction counts; the darker the colour, the more frequently two genomic loci were found proximal within a population of cells. An unmappable repetitive sequence region is shown in white. **(B)** Interactions at higher resolutions can be predicted using the HiP-HoP polymer simulation scheme (Rico et al., 2022). Interactions for the same region as above are shown binned at 3 kbp resolution; colour scale units are log simulated interaction counts. Here, the darker the colour, the more frequently two genomic loci were found proximal within a population of simulated chromatin configurations. **(C)** The simulated Hi-C is plotted again, but now the number of interaction counts is normalised to account for the effect of genomic separation, giving an “interaction enrichment” plot (darker colours indicate that interactions are enriched compared to what would be expected for a random pair of loci at the same genomic separation; see main text for details). Data are binned at 3 kbp resolution, and colour scale units are log_2_(observed simulated reads/expected simulated reads).

The HiP-HoP model uses the statistical physics of polymers to predict the chromatin configurations in a genomic region using only DNase-seq and ChIP-seq data from three histone modifications and CTCF. The model generates a population of simulated configurations from which Hi-C-like interaction information can be extracted. Three structure-driving mechanisms are included in the model: cohesin-CTCF loop extrusion, chromatin binding protein complexes driving regulatory element interactions and protein condensate formation, and a “heteromorphic polymer” accounting for variation of chromatin fibre properties. Figure 3 shows Hi-C data at a 10 kbp resolution for GM12878 cells (Rao et al., 2014), alongside our simulated Hi-C data at a 3 kbp resolution for the same cell line (Rico et al., 2022). This predicts that a region 3′ of *MYEOV* shows strong interactions with the promoters of *CCND1* and *LTO1*; furthermore, there is a “stripe” of interactions with both of these promoters extending downstream (i.e., 3′) from *MYEOV*. In Hi-C data, the level of interaction between any pair of loci strongly depends on their genomic separation (it decreases roughly as the inverse of genomic separation); it is therefore common to normalise for this effect. To achieve this an “expected” level of interaction for a given genomic separation is computed by finding the mean number of interactions across all pairs of loci with the given separation in the dataset; taking the observed number of reads at each point in a Hi-C map and dividing by this “expected” value, then yields a map of “interaction enrichment”. Completing this procedure for our simulated Hi-C gives an even more striking presentation of the prediction that *MYEOV* strongly interacts with *CCND1* and *LTO1* (bottom row in Figure 3). Further interrogation of the simulation reveals that (at least *in silico*) the stripe mainly arises due to loop extrusion by cohesin complexes which become halted on one side at the CTCF sites near *CCND1* and *LTO1* and drive looping from those genes towards *MYEOV*; in the model the strong peaks of interaction between DNase I hypersensitive sites within the regulatory element are driven by transcription factor complex binding.

The experimental data and the results from the simulations strongly suggest that the human *MYEOV-3*′*-putative enhancer* interacts with the proto-oncogene *CCND1* and *LTO1*. The ORF of *MYEOV* is found only in primates (Papamichos et al., 2015) but we do not know if the *MYEOV-3*′*-putative enhancer* originated around the same evolutionary time. Therefore, we decided to investigate the evolutionary conservation of the *MYEOV-3*′*-putative enhancer* and its interaction with *CCND1* in other species.

### 3.4 The *MYEOV-3*′*-putative enhancer* element in conserved in mammals and chicken

To test whether the *MYEOV*-3′-putative enhancer sequence was conserved in non-human primates, we performed a BLAST search using the *MYEOV* locus as a query to look for homologous sequences in six other primate species (Supplementary Figure S5). We discovered that not only was the coding sequence of *MYEOV* strongly conserved across all seven primate species considered, but there was also conservation of the sequence (∼1 kb) where the human *MYEOV-*3′-putative enhancer is located (Supplementary Figure S5). This conserved sequence aligns with enhancer chromatin states observed in humans (Supplementary Figure S5). Sequence conservation was not only seen in closely related species, such as chimpanzee and gorilla, but also in more distantly related species like squirrel monkeys (Supplementary Figure S5).

**FIGURE 4:**
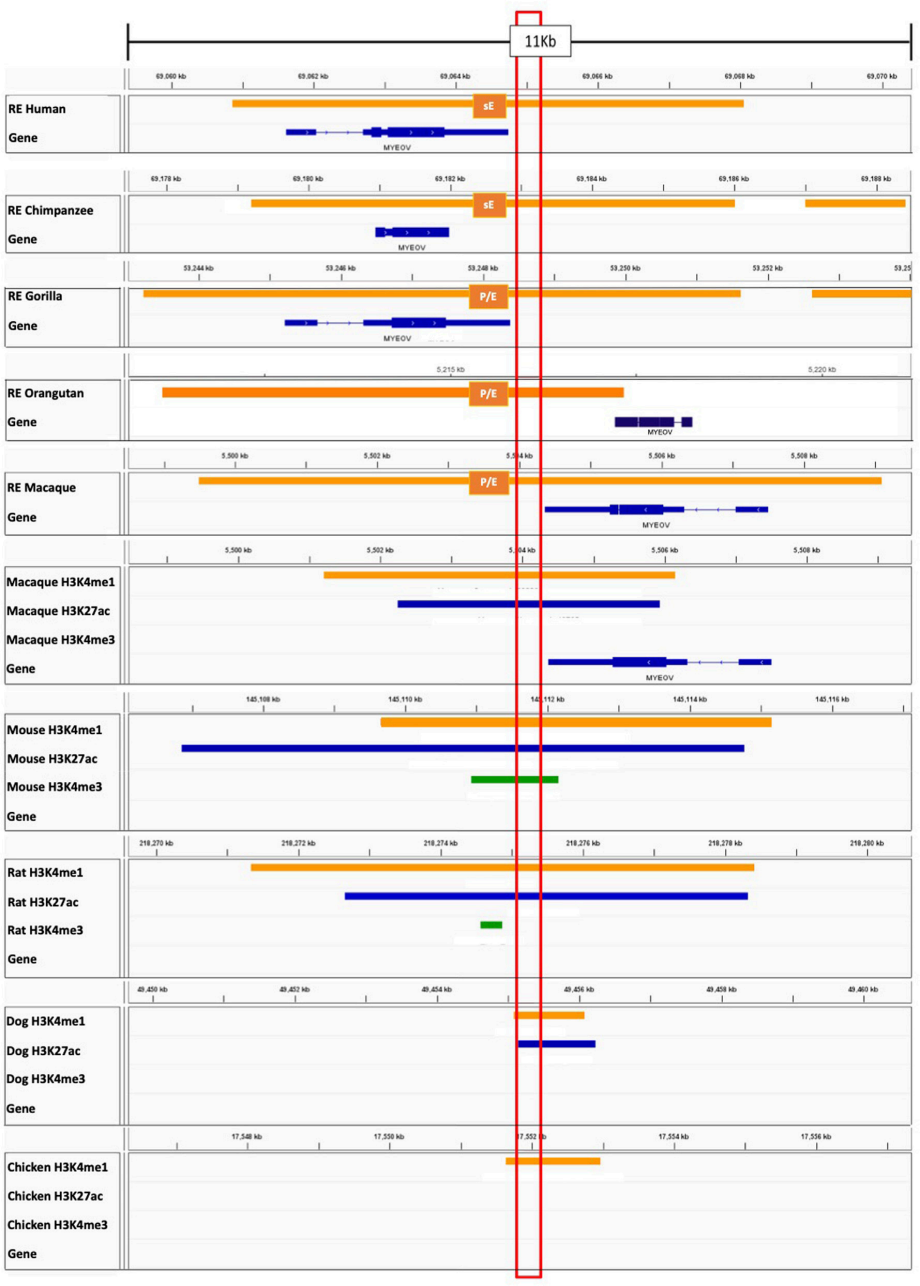
Homologous DNA regions in non-human species show enhancer-associated features. Top five tracks: Regulatory regions defined by ChromHMM in lymphoblastoid cells in human, chimpanzee, gorilla, orangutan, and macaque from García-Pérez et al. (2021). Abbreviation sE–Strong Enhancer, P/E−promoter/enhancer (where one replicate had an enhancer state and the other had a promoter state). Tracks below: ChIP-seq data for H3K4me1 (orange), H3K4me3 (green), H3K27ac (blue) derived from macaques, mice, rats, dogs and chicken (Kern et al., 2021; Roller et al., 2021). Individual sample replicates are combined into one track. The conserved region (the region with homologous sequence) is highlighted in the red box. *MYEOV*-3′-putative enhancer shaded in blue. Data is visualised on IGV.

These observations suggest that both *MYEOV* and its 3′ putative enhancer sequence are highly conserved outside of humans. To investigate if these regions also have enhancer activity in primates, we used the regulatory regions defined from a histone ChIP-seq dataset generated for lymphoblastoid cells of humans, chimpanzees, gorillas, orangutans and macaques (García-Pérez et al., 2021). We found that the entire *MYEOV* locus, including the homologous *MYEOV*-3′*-putative enhancer* region, is covered by strong enhancer or enhancer/promoter domains in the four non-human primates (Figure 4). This finding is particularly interesting since *MYEOV* is thought not to be transcribed in non-human primates (Papamichos et al., 2015), suggesting that this enhancer might be the only functional element in this region in these species despite the presence of ORFs. This is supported by data derived from orangutans, which are thought not to encode *MYEOV* due to a mutation leading to a premature stop codon (Papamichos et al., 2015), yet they still have an enhancer/promoter chromatin state present in the same region (Figure 4). This indicates that the *MYEOV-3*′*-putative-enhancer* element might have predated the development of an ORF in this region.

**FIGURE 5:**
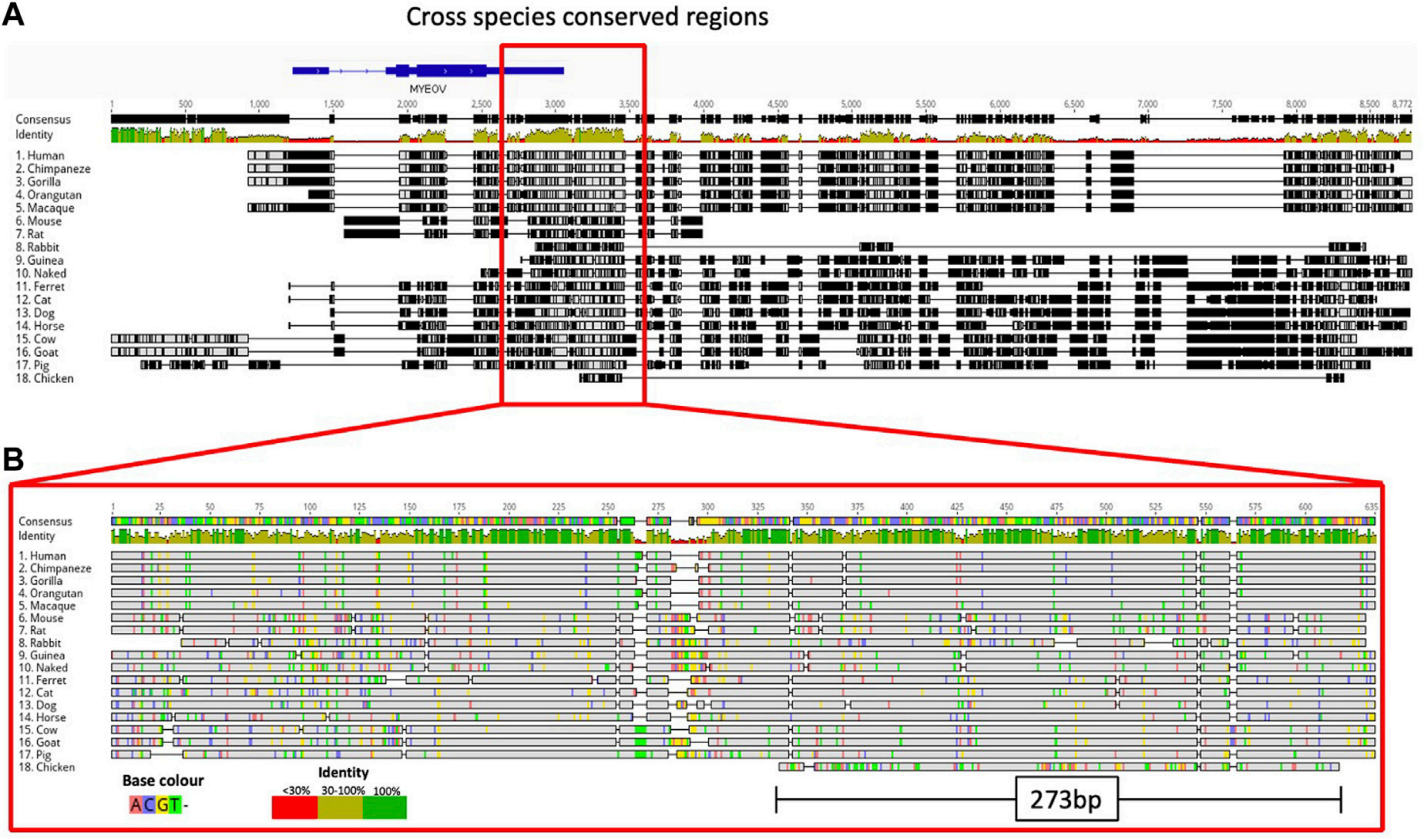
Multiple sequence alignment highlights conservation of *MYEOV-3*′*-putative enhancer* across multiple species. **(A)** Multiple sequence alignment illustrates the comparative analysis of the human *MYEOV-3*′*-putative enhancer* sequence with homologous sequences from 17 distinct species, identified through a BLAST search. The homologous sequences include 16 mammals, including four primates, and chicken. Sequence conservation is visualised using a colour-coded identity matrix, where darker colours signify higher sequence similarity. The consensus identity score is depicted, with yellow-green colours indicating conservation ranging from 50% to 100%. **(B)** Extraction and realignment of conserved regions, representing the largest contiguous block of identity scores between 50% and 100% present in all species. The realigned conserved region spans 635 bp for mammals and 273 bp for chicken. Identical residues are shaded in grey for clarity.

Next, we performed a comparative genomic analysis of additional species to determine if the *MYEOV-3′-putative enhancer* DNA sequence predated primates. We took the enhancer region defined by García-Pérez and co-workers (García-Pérez et al., 2021) as it encompassed both our defined *MYEOV-3′-putative enhancer* and the gene-body of *MYEOV*. BLAST searches across 17 mammalian and avian species, consistently observed BLAST hits ranging from 200-600 bp in a location similar to that between *CCND1* and *TPCN2* homologs across all species, except rabbits (Figure 5; Supplementary Figure S6; Supplementary Table S4) possibly due to the current version of the rabbit genome being highly fragmented (Bai et al., 2021). To determine the conservation of this enhancer element, we performed multiple alignments across all these species using the DNA sequences surrounding these BLAST hits. Expanding the search out to fishes, such as zebrafish, no sequence homology was found but we identified a homologous region in chicken, again located between the chicken *CCND1* and *TPCN2* homologs (Supplementary Figure S6). We were able to show conservation across 18 different species, with a 273 bp sequence being evolutionarily conserved up to and including chickens (Figure 5). This region aligns with the human *MYEOV-3*′*-putative enhancer* and overlaps with active regions called by ATAC-STARR-seq.

Using H3K27ac data across 20 mammalian species (Villar et al., 2015), we observed H3K27ac marking this conserved region in mice, rats, dogs, bovine and porcine epigenomes (Supplementary Figure S7). Using ChIP-seq data for H3K4me1, H3K4me3, and H3K27ac from ten mammalian species (Roller et al., 2021), and mice (Shen et al., 2012), we found conservation of the enhancer state across macaques, mice, rats, dogs and chicken with H3K4me1 and H3K27ac peaks surrounding the conserved region (Figure 5). Having observed sequence homology in chickens, we analysed ChIP-seq data for H3K4me3, H3K4me1, and H3K27ac derived from chicken liver tissue (Kern et al., 2021). H3K4me1 peaks were shown to surround the conserved region but no H3K27ac marks were detected, suggesting a potential poised enhancer state (Figure 5).

Taken together, these results suggest that *MYEOV-3′-putative enhancer* is possibly evolutionarily older than the *MYEOV* ORF. The patterns of histone marks in primates and non-primate mammals indicate a possible enhancer function of these homologous non-coding regions.

### 3.5 The mouse homolog of *MYEOV-3*′*-putative enhancer* interacts with *Ccnd1*

We have shown that the *MYEOV-3*′*-putative enhancer* is located between the corresponding homologs of *CCND1* and *TPCN2* in 16 out of the 17 species analysed. This finding suggests that this conserved enhancer may have regulatory connections with the *CCND1* homologs in these species (as we observed in humans). To test this hypothesis, we characterised the mouse homolog of the *MYEOV-3′-putative enhancer* in more detail.

We analysed data from Mouse ENCODE (Yue et al., 2014) for four different murine cell/tissue types to determine if this enhancer state was present in tissues other than the liver. We analysed H3K4me1 and H3K27ac data from CH12 cells (a lymphoma cell line used as a B cell model), lung tissue, liver tissue and bone marrow (Supplementary Figure S9). Similar to the equivalent human tissues, H3K4me1 and H3K27ac peaks covered the conserved region in all of these cell types (Supplementary Figure S9). We also noticed that downstream of the putative-enhancer, there is a CTCF site to which CTCF had bound in B cells (Supplementary Figure S9). This suggested that this enhancer might also be interacting with *Ccnd1* in mice, which is located in a similar location as seen in humans (Supplementary Figure S9).

**FIGURE 6:**
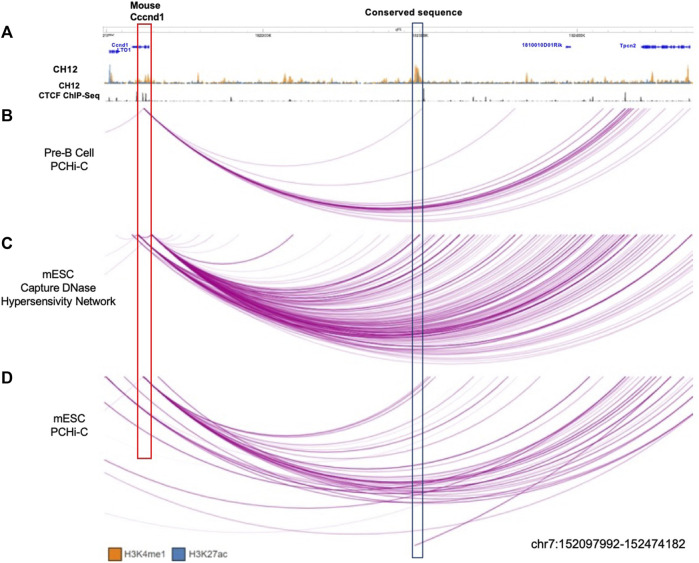
Interactions observed between conserved enhancer sequence and *Ccnd1*. **(A)** CH12 mouse lymphoma cell line ChIP-seq data for H3K4me1 (orange), H3K27ac (blue), and CTCF (black). **(B)** PCHi-C performed in pre-B cells in old mice (Koohy et al., 2018). **(C)** DNase capture Hi-C data from mESC (Joshi et al., 2015). **(D)** PCHi-C performed in mESC (Schoenfelder et al., 2015). The conserved enhancer region (the region with homologous sequence) is highlighted in the blue box. The purple arcs denote interactions between fragments. Data is visualised using the WashU Epigenome Browser.

To investigate whether this putative-enhancer region also targets *Ccnd1* in mice, we analysed PCHi-C data performed in both murine pre-B cells (Koohy et al., 2018) and mESCs (Schoenfelder et al., 2015) as well as DNase capture Hi-C data from mESCs (Joshi et al., 2015). We observed that in pre-B cells of old mice, *Ccnd1* interacts with this conserved putative-enhancer region (Figures 6A,B). This interaction can be also found in DNase capture Hi-C data performed in mESCs, which highlights interactions between open chromatin regions (Figure 6C). However, in PCHi-C data from mESCs (Schoenfelder et al., 2015), no interaction is seen with the evolutionarily conserved region, but an interaction is observed with a region just downstream of this element (Figure 6D). The difference in mESC capture data might be due to differences in the regions captured, as the *Ccnd1* bait is located at chr7:152,118,105-152,126,754 whereas in DNase capture Hi-C, the *Ccnd1* interacting site is at chr7:152,121,377-152,134,010.

In conclusion, these experimental data reveal that the putative-enhancer identified in mice shows chromatin interactions with *Ccnd1*, mirroring the interactions we observed between the *MYEOV-3*′*-putative enhancer* and *CCND1* in humans.

## 4 Discussion

An active enhancer chromatin state in B cells situated at the 3′ end of *MYEOV*, which we call the *MYEOV-3′-putative enhancer*, has previously been reported (Mikulasova et al., 2022). In this study we found that this enhancer chromatin state was exhibited across a diverse array of both healthy and disease-afflicted human cell types, thereby underscoring its possible role in regulating gene expression within varied cell types (Mikulasova et al., 2022).

Although the exact protein function of MYEOV remains elusive, ongoing studies highlight potential non-coding oncogenic functions at the transcript level. Overexpression of *MYEOV* has been associated with poor prognosis in different cancers (Janssen et al., 2002a; Janssen et al., 2002b; Leyden et al., 2006; Moss et al., 2006; Moreaux et al., 2010; Takita et al., 2011; Fang et al., 2019; Liang et al., 2020; Shen et al., 2021). Notably, transcripts of *MYEOV* possess oncogenic properties due to their capability to sequester miRNAs, thus preventing these molecules from targeting and repressing the expression of oncogenic factors (Fang et al., 2019).

Through an analysis of enhancer-associated histone marks (H3K4me1, H3K4me3, and H3K27ac) in the syntenic region, we unveiled a striking conservation of the putative enhancer chromatin state across mammals. *MYEOV* is a young protein-coding gene, with an ORF exclusive to primates but the DNA sequence of the *MYEOV-3*′*-putative enhancer* region exhibited conservation not only across mammals but also in chicken. Our results indicate that the histone marks associated with enhancer activity preceded the emergence of the ORF of *MYEOV* in primates. Another possibility is that these histone marks are associated with the expression of non-coding RNA transcripts.

Sequence homologs of this novel *MYEOV*-3′-*putative enhancer* were discovered through BLAST searches, where it was consistently situated adjacent to the homologs of *CCND1*. The synteny observed between *MYEOV* and *CCND1* across various species strongly suggests a functional relationship between these two elements. Furthermore, our research revealed that in human and murine cells (which lack an ORF, yet bear the enhancer marks) the *MYEOV*-*3*′*-putative enhancer* and *CCND1/Ccnd1* display interactions within the 3D genome. This suggests deep conservation (Wong et al., 2020) of this enhancer that extends beyond the ORF shared by most primates.

The *MYEOV-3*′*-putative enhancer* shows activity across a broad spectrum of human tissues, as indicated by the presence of H3K27ac. Consequently, we hypothesise that the *MYEOV-3*′*-putative enhancer* contributes to the regulation of *CCND1* expression. In non-pathogenic cells, the expression of *CCND1* is likely under the regulation of polycomb repressive complexes that exert their influence on its promoter region (Mikulasova et al., 2022; Rico et al., 2022). While the *MYEOV* gene is active and spatially proximate to *CCND1*, the expression of *CCND1* hinges on the removal of polycomb-mediated inhibition (Adhikari and Davie, 2020; Mikulasova et al., 2022). Conversely, in normal cells, the transcription of *MYEOV* remains inactive, largely attributed to DNA methylation (Fang et al., 2019; Liang et al., 2020). *CCND1* seems to have an association with more than one enhancer, as supported by the observation of multiple 3D genome interactions with regions bearing enhancer histone marks that extend beyond those linked with *MYEOV* (Rico et al., 2022). Future enhancer deletion/perturbation experiments using CRISPR (Kent et al., 2023) will help to prove whether the observed interactions between *CCND1* and *MYEOV-3*′*-putative enhancer* are indeed regulatory.

In the context of cancer, the insertion of super-enhancer elements from different chromosomes into the intergenic region flanked by the *MYEOV-3*′*-putative enhancer* and *CCND1* culminates in the emergence of an expansive active regulatory domain spanning the *CCND1* gene locus. This occurrence results in the displacement of repressive polycomb-associated signatures, ultimately paving the way for the activation of *CCND1* expression (Mikulasova et al., 2022). Intriguingly, this scenario concurrently activates *MYEOV*, as evidenced by the appearance of active promoter-associated marks (an H3K4me3 broad domain) over the *MYEOV*’s gene body (Mikulasova et al., 2022). This suggests that the introduction of these super-enhancer elements not only upregulates *CCND1* expression but also augments the transcriptional activity of *MYEOV* by creating a conducive chromatin environment through the acquisition of active promoter marks (Kent et al., 2023).

In cancer, documented cases of DNA amplification at 11q13, primarily likely selecting for *CCND1* proto-oncogene activation, often correspond to elevated *MYEOV* expression and an unfavourable prognosis (Fang et al., 2019; Liang et al., 2020). We speculate that during amplification events, duplicated genomic segments undergo the loss of their repressive epigenomic signatures, consequently enabling demethylation of *MYEOV*, and thereby leading to an overexpression of *MYEOV* RNA in cancer cells, and/or permitting the *MYEOV-3*′*-putative enhancer* to regulate *CCND1*, thereby boosting *CCND1* expression.

The concept of chromatin accessibility and enhancer characteristics that facilitate the birth of new genes has been proposed in prior research (Majic and Payne, 2020). Our findings align with this concept, effectively illustrating the potential of enhancers as a genomic environment that facilitates the emergence of new genes. Beyond its possible role in regulating *CCND1* and/or *LTO1*, the *MYEOV-3*′*-putative enhancer*’s significance in oncogenesis becomes all the more pronounced. It might actively contribute to oncogenic processes through DNA amplifications or chromosomal translocations of this locus containing both *CCND1* and *MYEOV-3*′*-putative enhancer* (Janssen et al., 2002b; Zhou et al., 2022), potentially allowing it to influence the expression levels of other proto-oncogenes (in addition to *CCND1*) that may end up in the proximity of the amplified copies, thereby expanding its impact on the dynamics of cellular function in cancer.

Finally, we would like to highlight that the conservation of the *MYEOV-3*′*-putative enhancer* element across species provides a unique opportunity for the development of animal models, including mouse or chicken, aimed at studying enhancer function and its interactions. While the creation of knockdown models of *MYEOV* in mice is impossible due to the gene’s absence, the preservation of the enhancer elements offers a promising avenue for functional exploration of the roles of the *MYEOV-3*′*-putative enhancer* in carcinogenesis.

## Data Availability

Publicly available datasets were analyzed in this study. The sources of the datasets can be found in Supplementary Table S1.

## Supplementary material

SUPPLEMENTARY FIGURE S1 NIH Roadmap data highlighting chromatin states surrounding *MYEOV*. **(A)** H3K4me1 (orange), H3K27ac (blue) and H3K4me3 (green) ChIP-seq data taken from NIH Roadmap data for GM12878, B cells, lung, liver and pancreatic tissues alongside DNase-seq data for GM12878 and B cells. Data was visualised on WashU genome browser. *MYEOV*-3’-putative enhancer is highlighted with an orange block. **(B)** Presence of H3K27ac peaks at *MYEOV’*s 3’ UTR region across 169 primary cell samples, tissues and cell lines. NarrowPeak ChIP-seq H3K27ac files were obtained from ENCODE and presence of peaks in chr11:69,297,041-69,298,854 was determined for each tissue. Data shown corresponds to Supplementary Table S2. Word clouds show biosample terms specific to groups with and without peaks, with darker coloured and larger words representing biosamples with more replicates.

SUPPLEMENTARY FIGURE S2 Interaction observed when *CCND1* is taken as viewpoint. Promoter capture Hi-C data across five different tissues/cell lines taken from Jung et al.; these being GM12878/GM19240 lymphoblastoid cell line, lung, liver and pancreatic tissues and H1-derived mesenchymal stem cells. *CCND1* was taken as bait and interactions were filtered to only show significant interactions over −log10(P-value) = 2 and normalised counts. Data was visualised on 3DIV.

SUPPLEMENTARY FIGURE S3 Interaction observed when *MYEOV* is taken as the viewpoint. Promoter capture Hi-C data across five different tissues/cell lines taken from Jung et al.; these being GM12878/GM19240 lymphoblastoid cell line, lung, liver,pancreatic tissues and H1-derived mesenchymal stem cells. *MYEOV* was taken as bait and interactions were filtered to only show significant interactions over −log10(P-value) = 2 and normalised counts. Data was visualised on 3DIV.

SUPPLEMENTARY FIGURE S4 Interaction observed when *LTO1/ORAOV1* is taken as bait. Promoter capture Hi-C data across five different tissues/cell lines taken from Jung et al.; these being GM12878/GM19240 lymphoblastoid cell line, lung, liver and pancreatic tissues and H1-derived mesenchymal stem cells. *ORAOV1* (*LTO1*) was taken as bait and interactions were filtered to show only significant interactions over −log10(P-value) = 2 and normalised counts. Data was visualised on 3DIV.

SUPPLEMENTARY FIGURE S5 BLAST Hit for *MYEOV* locus seen in seven primate species. Ensembl BLAST search was performed using the *MYEOV* locus as a query against seven primate species which included chimpanzee (*Pan troglodytes*), gorilla (*Gorilla gorilla*), orangutan (*Pongo abelii*), gibbon (*Nomascus leucogenys*), rhesus Macaque (*Macaca mulatta*), marmoset (*Callithrix jacchus*), and squirrel monkey (*Saimiri boliviensis*). Conserved sequences are shown as red rectangles with the percentage ID of BLAST hit determined by the shade. Chromatin data also shown from B cells with H3K4me1 (orange), H3K4me3 (green) and H3K27ac (blue) ChIP-seq data highlighted. *MYEOV*-3’-putative enhancer shaded in blue. Data was visualised on the Ensembl.

SUPPLEMENTARY FIGURE S6 Conservation of gene synteny surrounding BLAST sequence. Region which surrounds Ensembl BLAST top hit was located across five different species, these being human, dogs, cows, goats and chickens when *MYEOV* 3’UTR was used as the query sequence. Each BLAST hit is denoted with a red line. Data was visualised on the Ensembl.

SUPPLEMENTARY FIGURE S7 Conservation of H3K27ac signal across mammalian tissue. H3K27ac (blue) and H3K4me3 (orange) ChIP-seq data taken from liver tissue of six species including humans, mice, rats, dogs, cows and pigs (Villar et al. 2015). Also, H3K4me1 and H3K4me3 ChIP-seq data from mouse liver tissue (Shen et al. 2012). Conserved region highlighted in red is the homologous sequence determined from multiple sequence alignment. In each species, we indicate if the BLAST hit corresponded to the positive or negative strand of the species reference genome. Data was visualised on IGV.

SUPPLEMENTARY FIGURE S8 Comparing histone data from Roller et al. to Villar et al. ChIP-seq data for three mammalian species was taken from both Roller et al and Villar et al. This included H3K27ac (blue), H3K4me1 (orange) and H3K4me3 (green) for rats, dogs and pigs liver tissue. Conserved region highlighted in red is the homologous sequence determined from multiple sequence alignment. Data was visualised on IGV.

SUPPLEMENTARY FIGURE S9 Conservation of enhancer chromatin state across multiple mouse tissues. Top Panel: Highlights ChIP-seq for H3K4me1 (orange), H3K4me3 (green) and H3K27ac (blue) in CH12 mouse lymphoma cell line, lung, liver and bone marrow tissue in mice. Alongside, ChIP-seq data for CTCF in CH12 mouse lymphoma cell line. Bottom Panel: Show zoom in region surrounding conserved region which is highlighted in yellow and is the homologous sequence determined from multiple sequence alignment.

SUPPLEMENTARY TABLE S1 This table presents sources used for studying chromatin states and regulatory regions across different species and tissues. It contains sources of chromatin profiling data, including ChIP-seq and ATAC-seq, for multiple species and tissues. The data include information on histone marks (H3K4me1, H3K4me3, H3K27ac, H3K27me3, H3K36me3) and are sourced from diverse studies involving primates, other mammalian species, and chicken.

SUPPLEMENTARY TABLE S2 This table presents the results of an extensive analysis of the presence or absence of H3K27ac peaks within MYEOV’s 3'UTR region in a wide range of human tissues, cell lines, and cell types, shedding light on the tissues where this enhancer is active.

SUPPLEMENTARY TABLE S3 This table presents the data resources used to investigate the regulatory roles of MYEOV’s enhancer element in both human and mouse genomes and identification of gene targets of MYEOV’s enhancer. The table includes sources of RNAseq, ChIP-seq data for histone modifications (H3K4me1, H3K4me3, H3K27ac) and CTCF in multiple mouse tissues, including CH12 mouse B cell lymphoma cell line, adult liver cells, lung liver cells, and bone marrow. Additionally, it provides sources of human PCHi-C studies in specific tissues associated with cancer. The table also contains data on long-range chromatin interactions, ChIA-PET data, and CTCF data to explore potential mechanisms of enhancer-target promoter interactions and confirm topologically associated domain (TAD) structures.

SUPPLEMENTARY TABLE S4 This table presents DNA sequence conserved regions of MYEOV’s enhancer element across a diverse set of 17 species, pinpointed by BLASTN search, and multiple sequence alignments, are documented in this table.

SUPPLEMENTARY TABLE S5 This table presents a summary of the interactions observed by PCHi-Cdata between *MYEOV*, *LTO1* and *CCND1* highlighting the primary cell types, tissues and cell lines where the interactions were identified. Abbreviation. *put.* putative.
