# Supplementary figures and images for: Evolutionarily conserved enhancer-associated features within the MYEOV locus suggest a regulatory role for this non-coding DNA region in cancer
Source: Front Cell Dev Biol. 2024 Jul 30;12:1294510. doi: 10.3389/fcell.2024.1294510 (PMC11319300; doi:10.3389/fcell.2024.1294510)

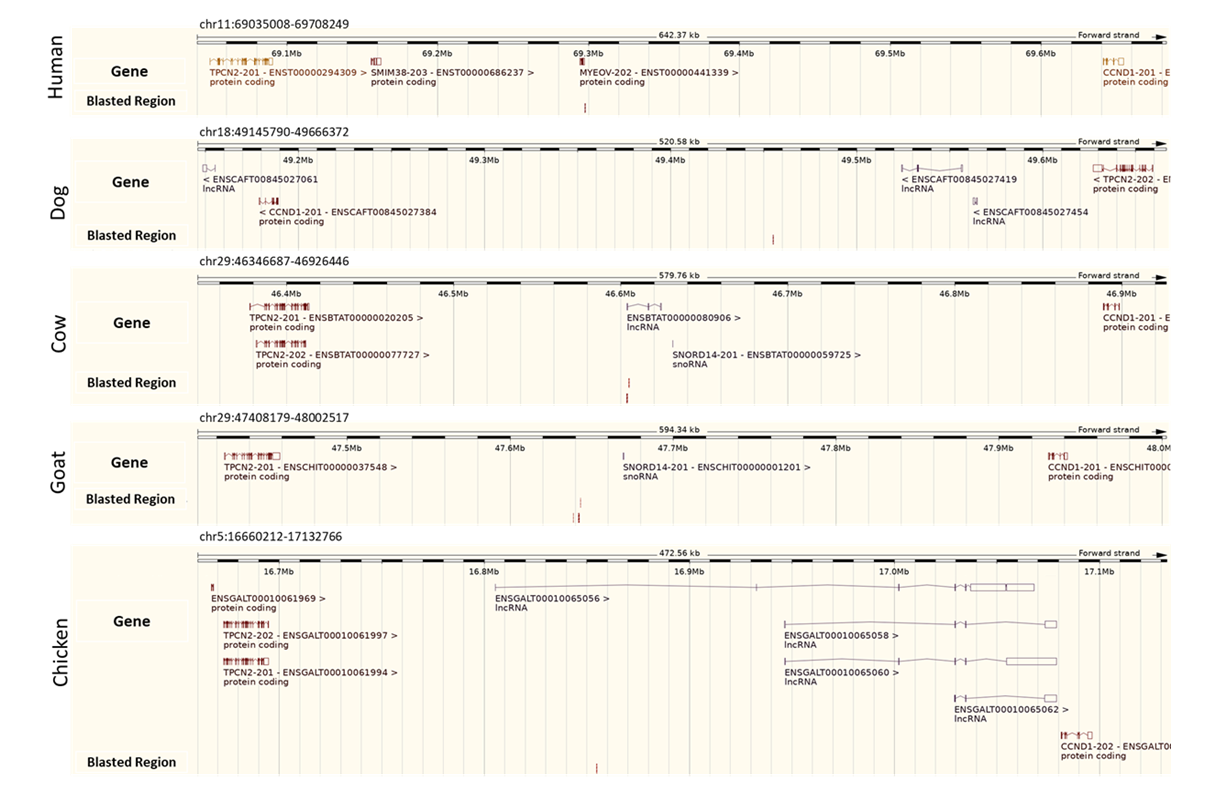

Supplement: Supplementary file 1 [file Image6.TIF]

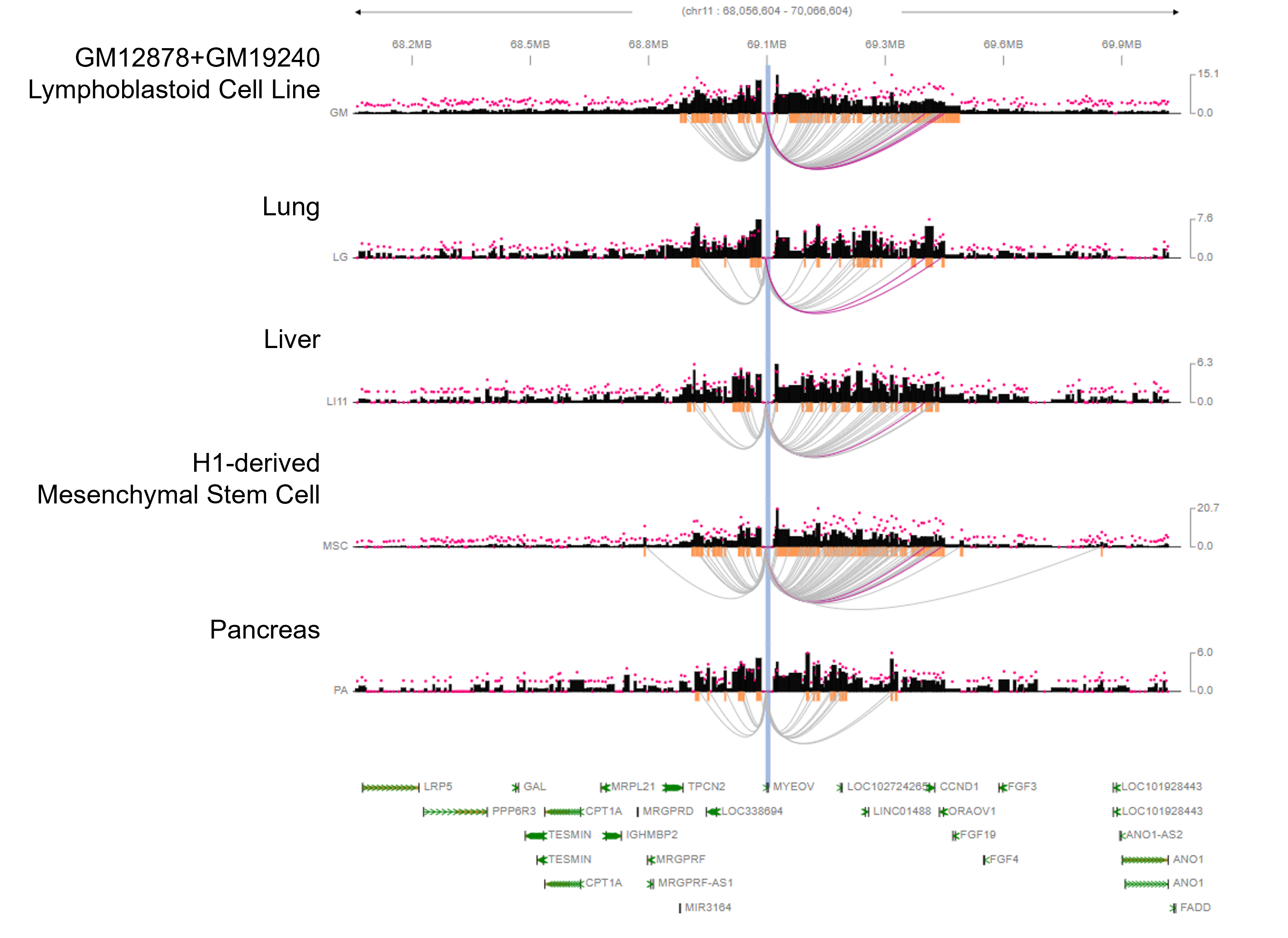

Supplement: Supplementary file 2 [file Image3.TIF]

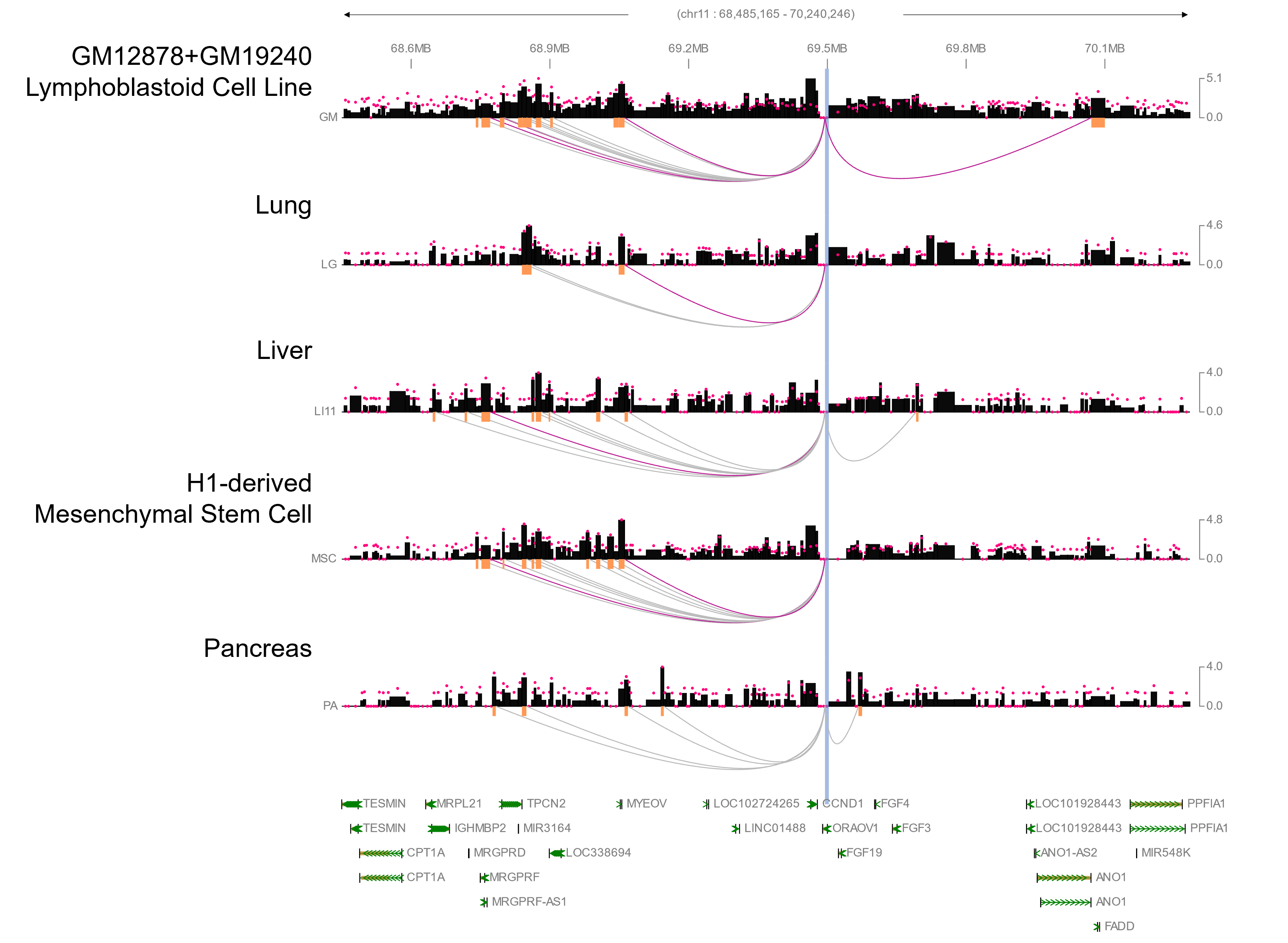

Supplement: Supplementary file 3 [file Image4.TIF]

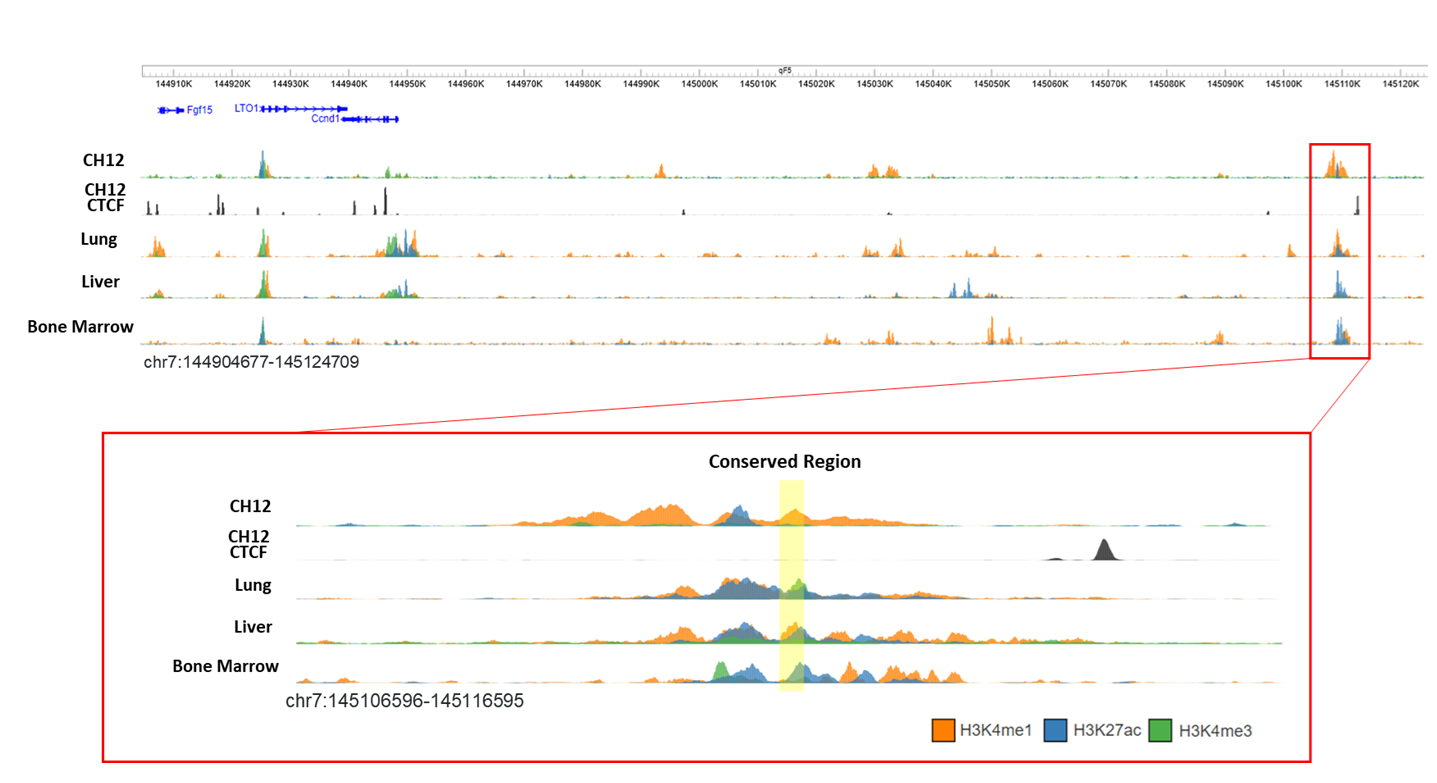

Supplement: Supplementary file 4 [file Image9.TIF]

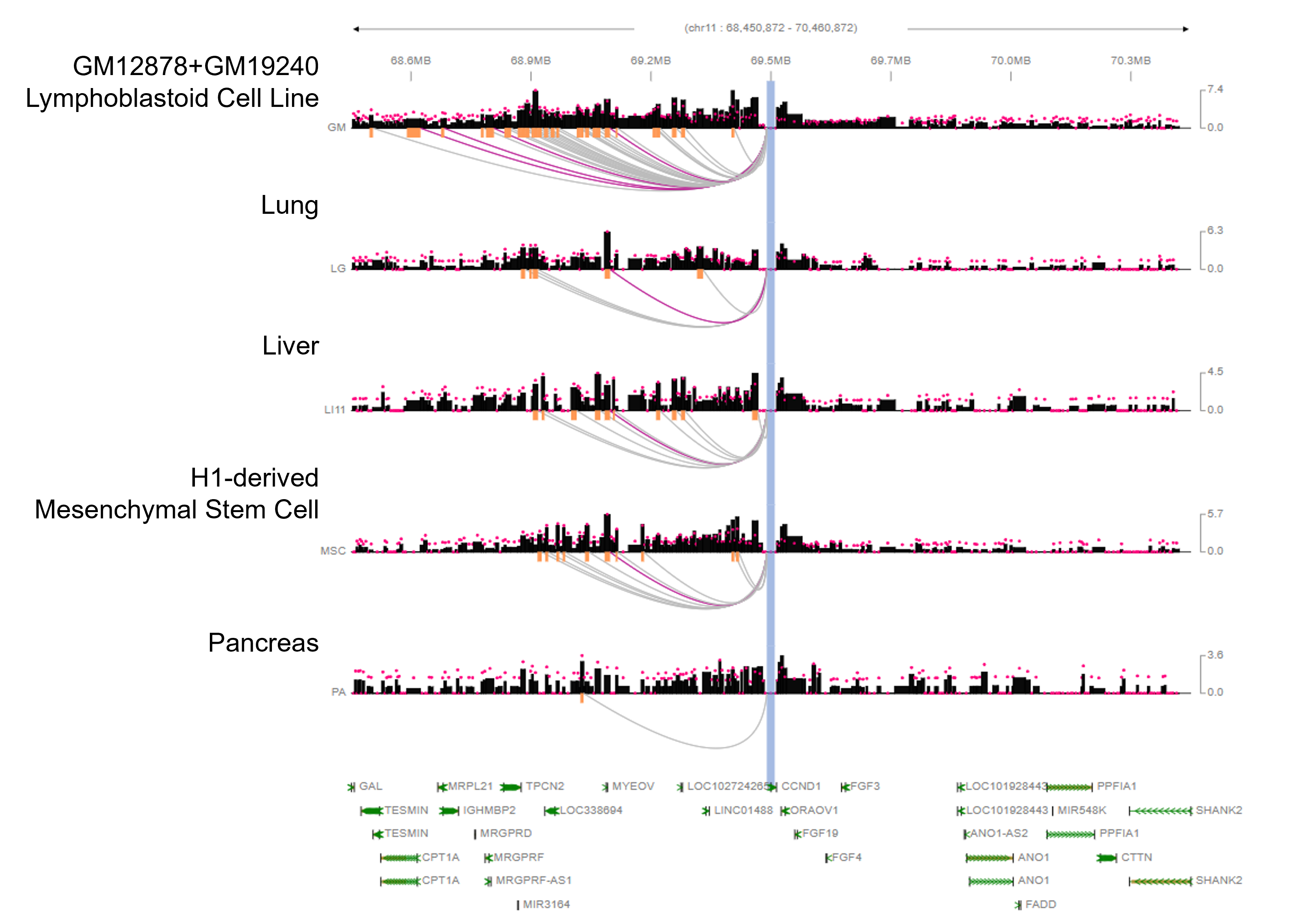

Supplement: Supplementary file 5 [file Image2.TIF]

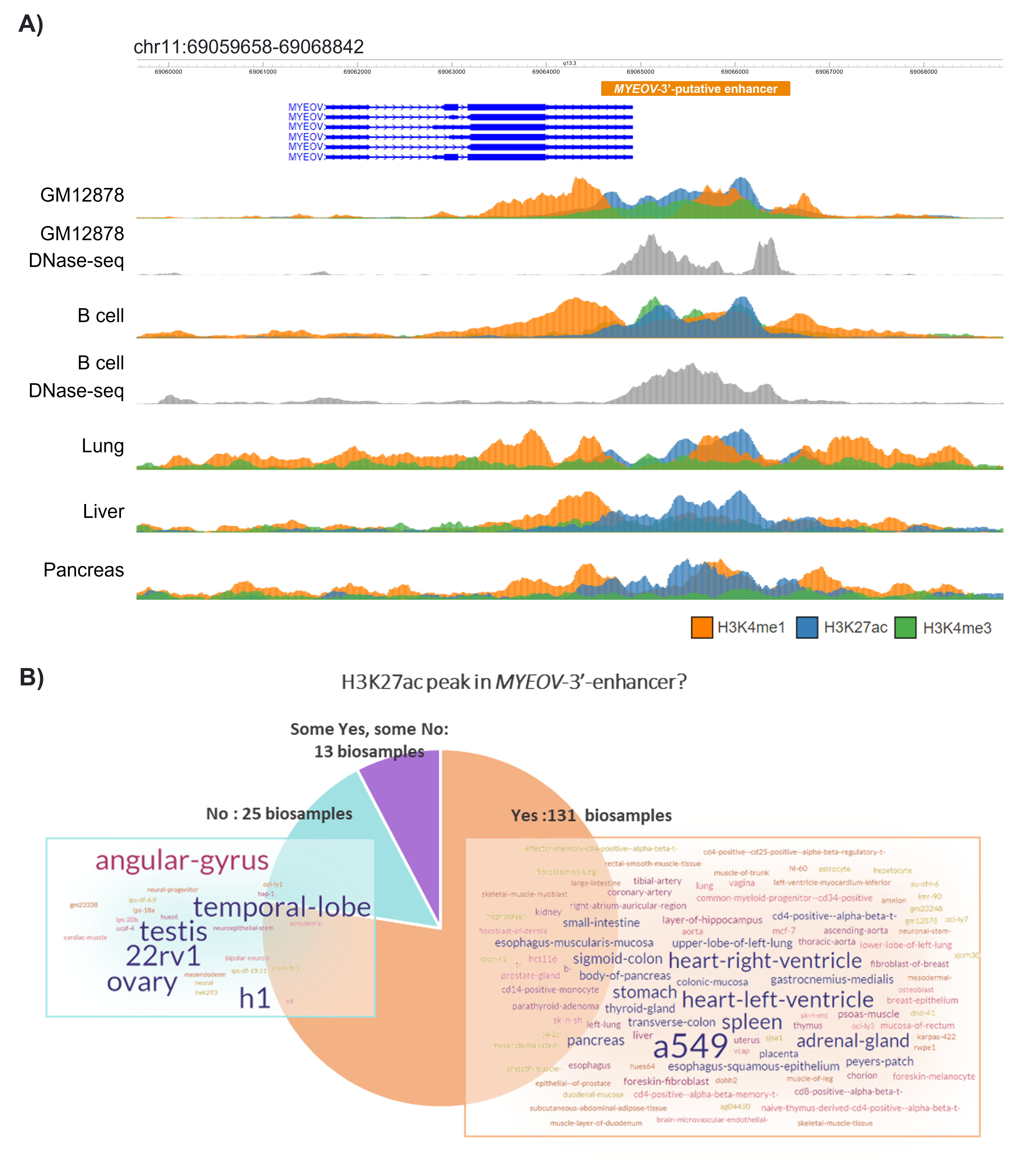

Supplement: Supplementary file 6 [file Image1.TIF]

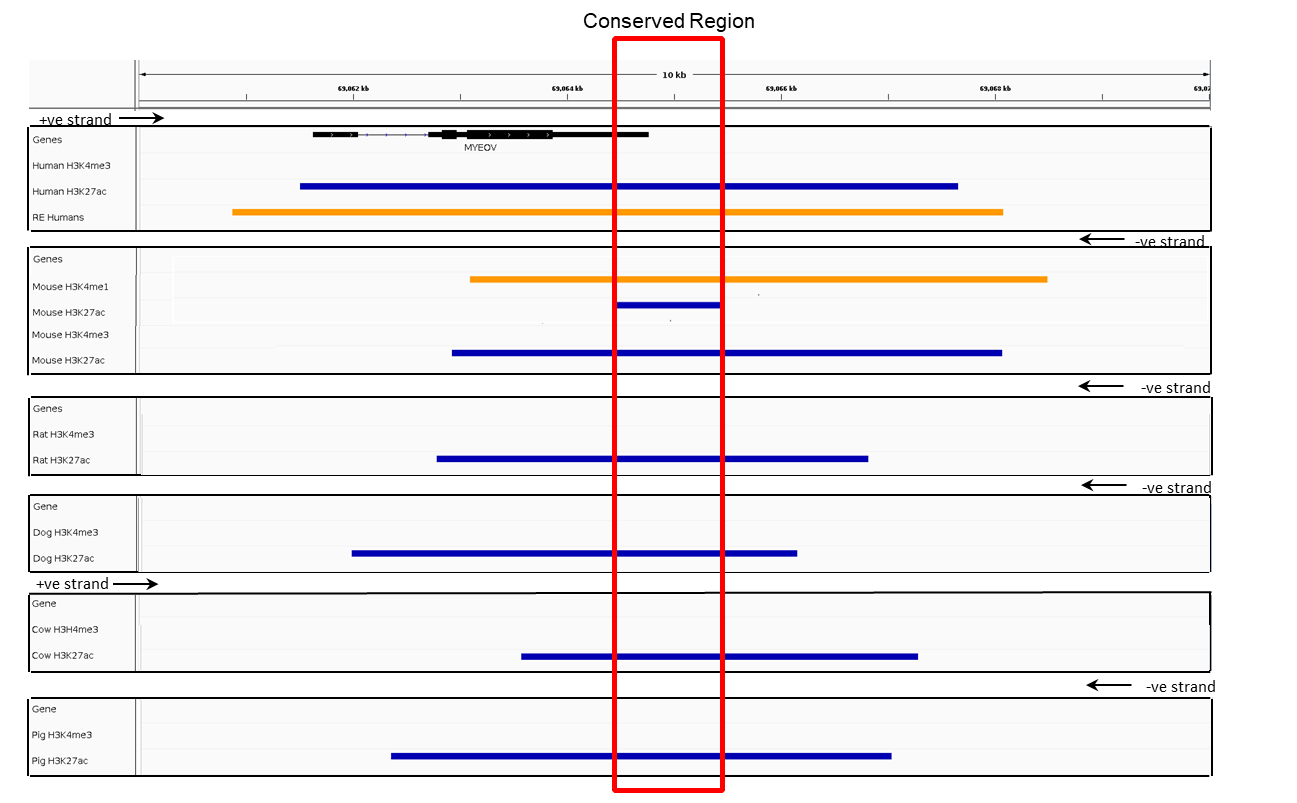

Supplement: Supplementary file 7 [file Image7.TIF]

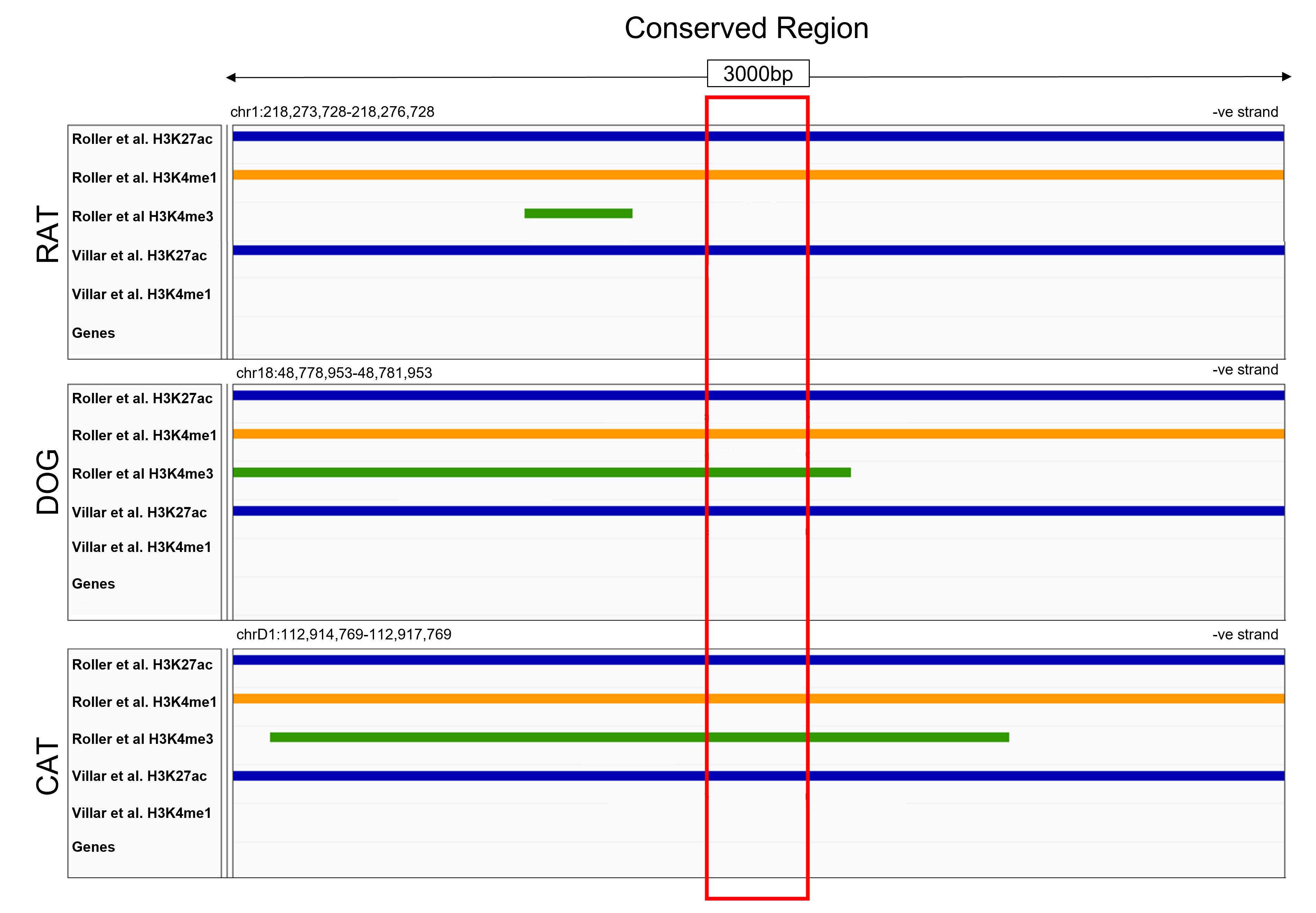

Supplement: Supplementary file 9 [file Image8.TIF]

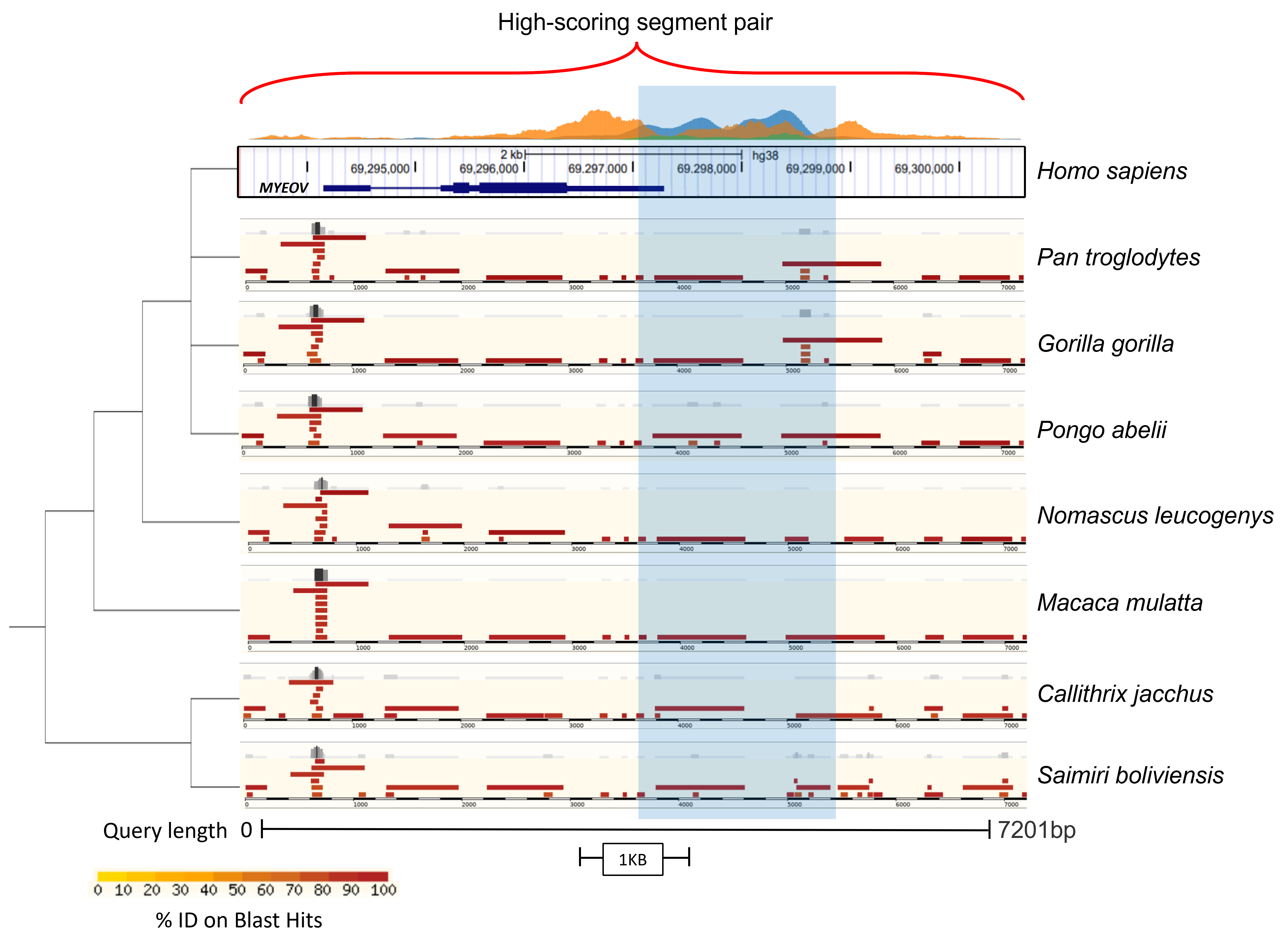

Supplement: Supplementary file 10 [file Image5.TIF]
